# Efficacy of 90Y ibritumomab-tiuxetan treatment in a case of resistant gastric MALT non-Hodgkin’s lymphoma

**DOI:** 10.3332/eCMS.2008.79

**Published:** 2008-03-13

**Authors:** PF Ferrucci, A Vanazzi, C Crosta, G Pruneri, C Grana, M Bartolomei, G Paganelli, G Martinelli

**Affiliations:** 1 Hematoncology Division, European Institute of Oncology, Via Ripamonti 435, 20100 Milan, Italy

## Abstract

Treatment modalities for resistant/relapsing gastric mucosa associated lymphoid tissue (MALT) non-Hodgkin’s lymphoma (NHL) are not yet well standardized. In the past, most patients were treated surgically with a gastrectomy, while, more recently, radiotherapy and systemic approaches (chemotherapy and immunotherapy) have been used with improving results.

Here, we report the case of a patient affected by MALT NHL resistant to antibiotics, chemotherapy and immunotherapy, who achieved a durable complete remission after radio-immunotherapy treatment with Zevalin (^90^Y ibritumomab-tiuxetan), administered in a single-standard dose. This observation must be confirmed on a larger series but suggests that radio-immunotherapy may be a valid approach in treating relapsing MALT NHL patients, or those resistant to conventional therapies, so avoiding more aggressive and toxic approaches.

## Introduction

Mucosa associated lymphoid tissue (MALT) lymphoma is usually associated with *Helicobacter pylori* (HP) infection, and its eradication using antibiotics and proton pump inhibitors is considered the treatment of choice, inducing tumour regression in approximately 70% of cases [1,2].

Surgery, most frequently adopted in the past for resistant or relapsing MALT NHL patients, has been progressively replaced by radiotherapy (RT) in the attempt to reduce physiological impairments while maintaining similar results in disease control [3,4]. RT is now considered the first option in many countries, including the United States as it is has a 90% complete response rate and 77% disease-free survival. Reported toxicity is generally mild although it can be associated with a potential risk of acute and chronic side effects [4]. However, this modality of treatment is not always feasible due to logistical and technical problems, which may justify exploring alternative options such as radio-immunotherapy (RIT).

Preliminary results suggest that immunotherapy with anti-CD20 monoclonal antibodies (Moab) may be effective in treating marginal zone non-Hodgkin’s lymphomas with nodal or extra nodal presentation, including gastric MALT ones [5,6].

RIT is a new area of medicine that combines radiation and immune therapy, using mechanisms in which radiolabelled monoclonal antibodies are used as ‘guided missiles’ to specifically target tumour cells. In fact, the specificity of the antibody for a receptor on the tumour cell directs the toxic effect of radiation precisely to the tumour site [7]. Once bound to the target cells, the radiolabelled antibody delivers radiation, which will destroy the cancer cell. A benefit from this type of radiation could be a ‘crossfire effect’ in which cells not directly targeted by the Moab, but close to it, may also receive a radiation dose and be destroyed. Moreover ^90^Y, which is a pure beta-emitter, penetrates tissues up to 11 mm with a median range of 5 mm sparing undesirable toxicity while maintaining efficacy. These characteristics make such an option very promising in treating diseases requiring a combination of systemic and local therapeutical effects.

Zevalin (ibritumomab-tiuxetan) is a ^90^Y radiolabelled monoclonal antibody, which targets the CD20 receptor on both normal and malignant mature b-cells and has been demonstrated to be active in follicular and diffuse large B-cell NHL, also in cases resistant or refractory to rituximab [8–10]. Zevalin attacks both normal and abnormal (malignant) B-cell lymphocytes. However, the body quickly replaces any normal white blood cells that are damaged so the risk of side effects from this is very small.

Here, we report our experience using Zevalin on a gastric MALT NHL patient resistant to antibiotics chemotherapy and immunotherapy.

This observation should be further investigated but suggests that RIT may be a valid approach in treating MALT NHL patients who are relapsing or resistant to conventional therapies with reduced toxicity compared to standard approaches.

## Case report

A 35-year-old Caucasian man was admitted to our hospital with a symptomatic resistant gastric MALT NHL in July 2003. He had a history of stage IEA (Ann Arbour staging stage I for Lugano staging system for gastrointestinal lymphomas) [11] gastric MALT NHL previously diagnosed in February 2001. At that time, due to the presence of HP infection, he received amoxicillin omeprazole and clarithromycin as eradication treatment. Two months later, an upper gastrointestinal endoscopy (UGE) with multiple gastric biopsies revealed a complete clinical and pathological response.

In February 2002, the patient experienced a local relapse without evidence of HP and was therefore treated with three cycles of standard cyclophosphamide, oncovin, doxorubicin, prednisone chemotherapy, achieving a minimal response with disappearance of all symptoms. Three additional cycles were performed but final evaluation revealed the persistence of microscopic disease. CT scans excluded distant disease. The patient was asymptomatic and declined further treatment for 14 months but, in May 2003, an additional course of antibiotics was suggested because of the onset of local symptoms. No clinical and pathological benefit was attained, and the patient was therefore considered for a total gastrectomy, which he declined.

In July 2003, he received the anti-CD20 monoclonal antibody rituximab in a four-week schedule as a part of a phase II study for patients affected by resistant or relapsing gastric MALT NHL. After two months, a UGE revealed microscopic persistence of lymphoma again with the disappearance of related symptoms. A maintenance treatment with four additional monthly doses of rituximab was delivered until December 2003, but the presence of MALT lymphoma was confirmed by multiple gastric biopsies. Due to asymptomatic disease, we decided to observe the patient with close follow-up.

In May 2004, the patient became newly symptomatic and the UGE documented a disease progression (Figures 2A–B).

**Figure 1:**
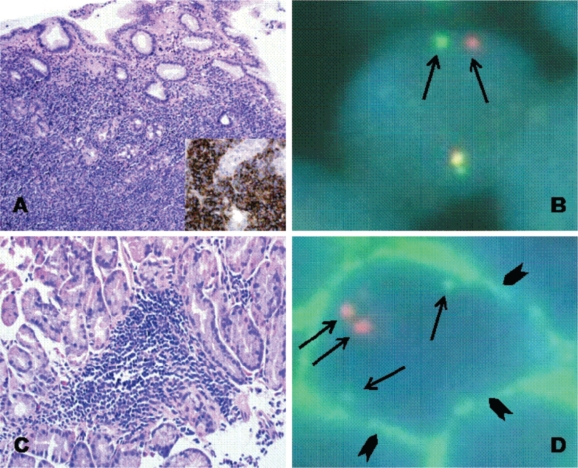
At diagnosis the gastric biopsy is heavily infiltrated by a dense population of small and medium-sized lymphocytes, which form lympho-epithelial lesions with the gastric glands and are immunoreactive for CD20 (A). Interphase FISH, using the LSI MALT1 Dual Color, Break Apart DNA Probe, showed split signals in most of the nuclei analysed; a pattern consistent with the occurrence of an 18q21 locus translocation involving the MALT1 gene (B). After therapy, the gastric biopsies showed a few, small, well-demarcated lymphoid nodules (C), containing rare lymphocytes with membranous immunofluorescence for CD20; two discrete API2 (green fluorescence) and MALT1 (red fluorescence) signals can be observed suggesting the absence of the t(1118)(q21q21) translocation and therefore of minimal neoplastic involvement (D).

Pathological evaluation showed gastric mucosa heavily infiltrated by a dense population of small and medium-sized lymphocytes immunoreactive for CD20, which formed lympho-epithelial lesions within the gastric glands (Figure 1A). Interphase FISH using the LSI^®^ MALT1 Dual Color, Break Apart Probe (Vysis, Inc., Downers Grove, IL, USA) showed split signals in most of the nuclei analysed. This pattern was consistent with the occurrence of an 18q21 locus translocation, involving the MALT1 gene (Figure 1B), that was further characterized as at (1118)(q21q21) by using the LSI^®^ API2/MALT1 Dual Fusion Translocation probe (Vysis, Inc., Downers Grove, IL, USA) [6].

Endoscopic ultrasonography (EUS) showed hypoecogenic gastric wall pachynsis at the corresponding lesion, while total body CT scans and bone marrow biopsy excluded other disease localizations.

The patient was informed about the possibility of receiving RT on the stomach as a standard treatment after failure of the previous approaches, but he was concomitantly proposed to participate in a phase II study of RIT with Zevalin carried out in our institute for patients with resistant/refractory MALT NHL. Due to logistical problems and considering the possibility of performing RT even after Zevalin treatment, the patient signed the informed consent and was enrolled in the RIT study.

Before RIT administration, blood count was normal, hepatic renal and cardiac functions (evaluated by serum chemistry electrocardiography and echocardiography) showed no impairments.

Treatment with ^90^Y ibritumomab tiuxetan (Zevalin) was preceded by rituximab 250 mg/sqm on day 0 and 8. On day 8, Zevalin was administered at the dose of 0.4 mCi/kg (maximum dose 32 mCi) with a 10-minute intravenous injection for therapy. ^111^In ibritumomab tiuxetan imaging dosimetry was not performed since it is not mandatory.

Clinical and haematological evaluations were performed weekly for 12 weeks, then every two months for six months and thereafter once every six months. If the platelet level dropped below 30 × 10^9^/L, then the platelet count was performed three times a week for as long as it was below this level. Serum chemistry was monitored monthly for six months and then every six months.

Zevalin treatment induced a transient and reversible haematological toxicity characterized by grade III thrombocytopenia, grade IV neutropenia and grade I anaemia (NCI scale). Neither platelet nor red blood cell transfusions were necessary. Platelet and neutrophil count nadirs were achieved after five and six weeks from Zevalin administration and recovered in one and two weeks, respectively. Other non-haematological toxicity was absent.

**Figure 2:**
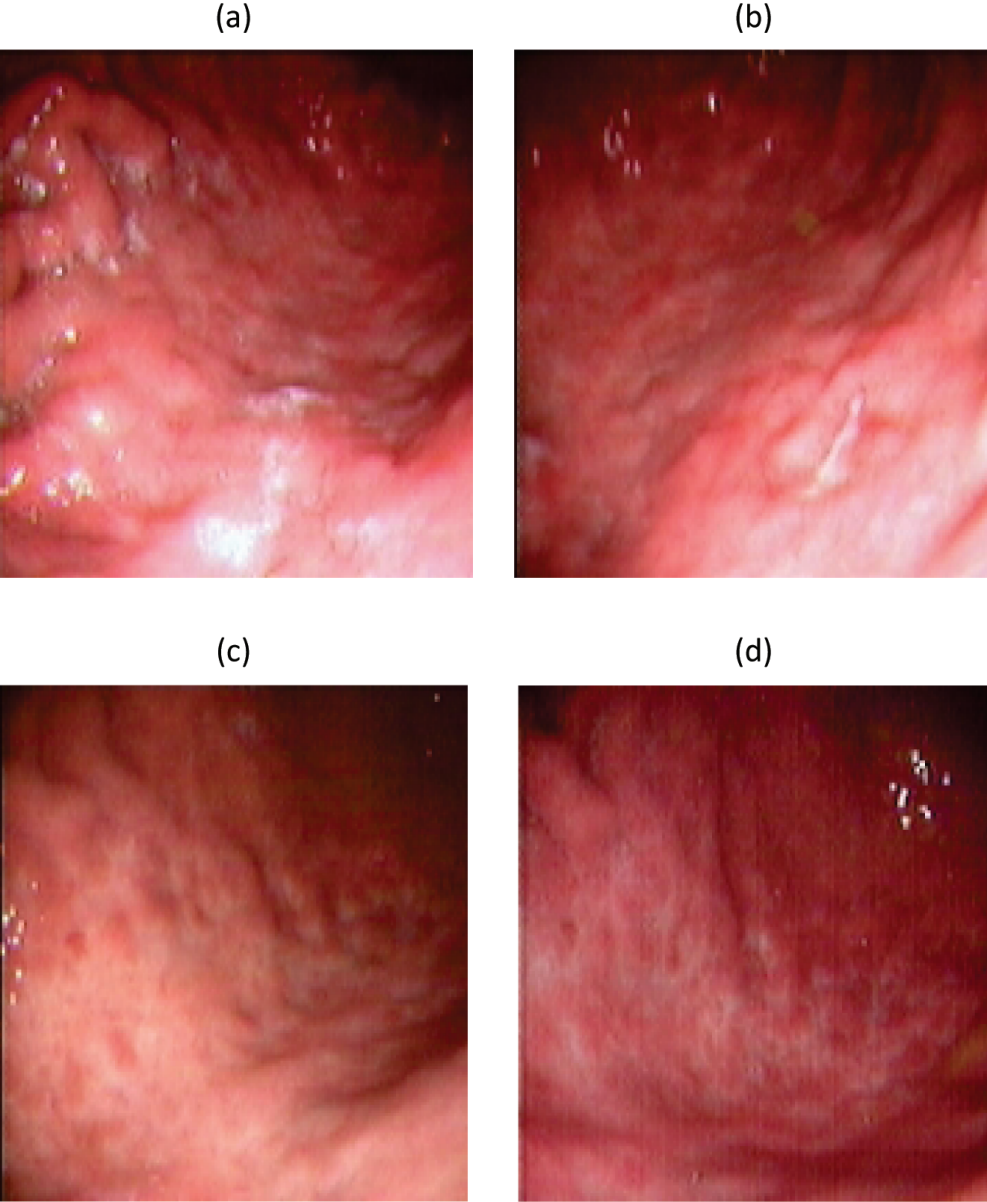
Upper Gastrointestinal Endoscopy. During endoscopic follow-up, a macroscopic recurrence of the lesions of the gastric greater curvature was observed, showing cobblestone appearance and a small 5 mm ulceration of the mucosa. Biopsies were positive for disease (A and B). Six months after radioimmunotherapy an upper gastrointestinal endoscopy showed chronic atrophic gastritis. Biopsies were negative for disease (C and D).

Four different endoscopic evaluations (UGE with multiple gastric biopsies and EUS) and total body CT scans were performed at two, four, six and 12 months from Zevalin administration, showing a clinical and pathological complete response (Figures 2C–D). In particular, gastric biopsies were characterized by small lymphocytes and plasma cells isolated in the *lamina propria* or forming small well-demarcated nodules (Figure 1C), with a normal pattern of the MALT1 and API-2 genes by interphase dual colour FISH (Figure 1D).

## Discussion

The optimal treatment for MALT NHL has not yet been defined. Literature data are not exhaustive since studies often refer to retrospective series of patients not uniformly staged or treated.

HP eradication with antibiotics is an effective first-line treatment but a strict endoscopic follow-up is recommended [12–21]. When first-line antibiotics fail or in absence of HP infection, a second line of antibiotics can be attempted, but the chance of response is significantly reduced.

Surgery, chemotherapy and RT alone or in combination are the treatments most frequently proposed to patients with resistant/relapsing gastric MALT NHL.

Surgery has been questioned in the last few years in favour of more conservative approaches. A total gastrectomy offers good chances of resolution but severely impairs quality of life.

Chemotherapy has never been adequately evaluated in gastric MALT lymphomas as it is not usually administered after surgery or radiotherapy [22].

Nowadays, RT seems to be the best therapeutic option for gastric MALT NHL. Tsang *et al* recently reported that up to 90% of the patients receiving RT alone achieved a complete response with an EF and OS of 77% and 98%, respectively, at five years [4]. Although RT may be proposed as the treatment of choice in MALT lymphoma patients not responding to eradication therapy, this series of patients was heterogeneous for the localization of the MALT lymphoma. In a previous study by Schechter *et al* [23], 17 patients with stage I–II gastric MALT lymphoma without evidence of HP infection or with persistence of lymphoma after eradication therapy, received radiation alone (1.5 Gy fractions in four weeks to the stomach, and the adjacent lymph nodes with a median total dose of 30 Gy) with 100% pathological CR and 100% EF survival at a median follow-up of 27 months.

Nevertheless, RT is a relatively complex procedure requiring a specific expertise not always available in small hospitals. Patients undergoing RT have to move to comprehensive cancer centres and often have to find alternative accommodation for long periods. Moreover, RT may induce acute and chronic side effects independent of administered dose.

Considering the indolent nature of MALT disease and the little differences in terms of OS or EFS reported between patients treated with various combinations of surgery, radiotherapy and chemotherapy [24–27], new therapeutic options less toxic or more compliant are worthwhile.

The anti-CD20 Moab (rituximab) has been convincingly effective in treating gastric and extra-gastric MALT NHL [5,6] but rarely obtaining durable responses when used as a single agent. Recently, preliminary data regarding the clinical activity of rituximab in the resistant or refractory gastric MZ NHL patient has been published. An objective response was observed in 77% of patients (with 31% of CR). The *t*(1118)(q21q21) translocation was not a predictive marker for response or subsequent relapse.

The use of radio-labelled Moabs may have a significant impact on the treatment of gastric MALT NHL. RIT allows specific cells to be targeted by Moab labelled with a radionuclide. The rationale for the use of RIT principally consists in the opportunity to target radiation directly to the tumour bulk, avoiding the exposure of other tissues. In particular, ^90^Y ibritumomab tiuxetan (Zevalin) is a murine parent of the anti-CD20 Moab (rituximab), conjugated to a beta emitter ^90^Y. It has been convincingly reported to be active in follicular and diffuse large B-cell NHL [7], but there is still very little published data on MALT NHL.

Witzig *et al* [10] recently reported four cases of MALT lymphoma, one of them with a gastric localization treated with a single-standard dose of Zevalin. All four patients had previously received chemotherapy and three of them received radiotherapy. The patient with gastric MALT NHL also had bone marrow involvement and obtained a PR with a time to progression of 19 months. The other three had lung/parotid skin and axilla localization. All of them obtained a complete response.

Our findings suggest a possible role of RIT in patients affected by gastric MALT NHL heavily pre-treated and resistant to immunotherapy and standard chemotherapy. We confirm and expand the preliminary results reported by Witzig *et al* [10].

Even though the prognosis of patients with MALT NHL is often good regardless of treatment, the possibility for alternative, less toxic treatments should always be considered. RIT could also be offered to those patients who have previously received RT or who are not suitable to receive it for various reasons. The toxic effects of RIT are limited, prevalently haematological, transient and reversible, even in a high activity setting, given peripheral stem-cell support [28]. The approach presented here needs further investigation and for this reason a phase II study is currently ongoing in our institute, delivering Zevalin at conventional dosage in resistant/refractory gastric MALT NHL patients.

## References

[1] Bayerdörffer E, Neubauer A, Rudolph B (1995). MALT Lymphoma Study Group. Lancet.

[2] Ferrucci PF, Zucca E (2007). Primary gastric lymphoma pathogenesis and treatment: what has changed over the past 10 years?. Br J Haematol.

[3] Schechter NR, Portlock CS, Yahalom J (1998). Treatment of mucosa-associated lymphoid tissue lymphoma of the stomach with radiation alone. J Clin Oncol.

[4] Tsang RW, Gaspodarowicz MK, Pintilie M, Wells W (2003). Localized mucosa-associated lymphoid tissue lymphoma treated with radiation therapy has excellent clinical outcome. J Clin Oncol.

[5] Conconi A, Martinelli G, Thieblemont C, Ferreri AJ (2003). Clinical activity of rituximab in extranodal marginal zone B-cell lymphoma of MALT type. Blood.

[6] Martinelli G, Laszlo D, Ferreri AJ, Pruneri G (2005). Clinical Activity of Rituximab in Gastric Marginal Zone Non-Hodgkin’s Lymphoma Resistant to or Not Eligible for Anti-Helicobacter Pylori Therapy. J Clin Oncol.

[7] Cheson BD (2003). Radioimmunotherapy of non-Hodgkin lymphomas. Blood.

[8] Witzig TE, Gordon LI, Cabanillas F (2002). Randomized controlled trial of Yttrium-90-labeled ibritumomab tiuxetan radioimmunotherapy versus rituximab immunotherapy for patients with relapsed or refractory low-grade follicular or transformed B-cell non-Hodgkin’s lymphoma. J Clin Oncol.

[9] Morschhauser F, Huglo D, Martinelli G, Paganelli G (2004). Yttrium-90 Ibritumomab Tiuxetan (Zevalin) for Patients with Relapsed/Refractory Diffuse Large B-Cell Lymphoma Not Appropriate for Autologous Stem Cell Transplantation: Results of an Open-Label Phase II Trial. Blood.

[10] Witzig TE, Gordon LI, Emmnouilides C, Wiseman GA, Dimitrov G, Ding E, Lee DD, White CA (2001). Safety and efficacy of Zevalin in four patients with Mucosa Associated Lymphoid Tissue (MALT) Lymphoma. Blood.

[11] Rohatiner A, d’Amore F, Coiffier B (1994). Report on a workshop convened to discuss the pathological and staging classifications of gastrointestinal tract lymphoma. Ann Oncol.

[12] Wotherspoon AC, Doglioni C, Diss TC (1993). Regression of primary low-grade B-cell gastric lymphoma of mucosa-associated lymphoid tissue type after eradication of Helicobacter pylori. Lancet.

[13] Roggero E, Zucca E, Pinotti G (1995). Eradication of Helicobacter pylori infection in primary lowgrade gastric lymphoma of mucosa-associated lymphoid tissue. Ann Intern Med.

[14] Bayerdörffer E, Neubauer A, Rudolph B (1995). Regression of primary gastric lymphoma of mucosa-associated lymphoid tissue type after cure of Helicobacter pylori infection: MALT Lymphoma Study Group. Lancet.

[15] Montalban C, Manzanal A, Boixeda D (1997). Helicobacter pylori eradication for the treatment of low-grade gastric MALT lymphoma: follow-up together with sequential molecular studies. Ann Oncol.

[16] Steinbach G, Ford R, Glober G (1999). Antibiotic treatment of gastric lymphoma of mucosa-associated lymphoid tissue: an uncontrolled trial. Ann Intern Med.

[17] Sackmann M, Morgner A, Rudolph B (1997). Regression of gastric MALT lymphoma after eradication of Helicobacter pylori is predicted by endosonographic staging: MALT Lymphoma Study Group. Gastroenterology.

[18] Weston AP, Banerjee SK, Horvat RT, Zoubine MN, Campbell DR, Cherian R (1999). Prospective long-term endoscopic and histologic follow-up of gastric lymphoproliferative disease of early stage IE low-grade B-cell mucosa-associated lymphoid tissue type following Helicobacter pylori eradication treatment. Int J Oncol.

[19] Savio A, Franzin G, Wotherspoon AC (1996). Diagnosis and posttreatment follow-up of Helicobacter pylori-positive gastric lymphoma of mucosa-associated lymphoid tissue: histology polymerase chain reaction or both?. Blood.

[20] Nobre-Leitao C, Lage P, Cravo M (1998). Treatment of gastric MALT lymphoma by Helicobacter pylori eradication: a study controlled by endoscopic ultrasonography. Am J Gastroenterol.

[21] Neubauer A, Thiede C, Morgner A (1997). Cure of Helicobacter pylori infection and duration of remission of low-grade gastric mucosa-associated lymphoid tissue lymphoma. J Natl Cancer Inst.

[22] Hammel P, Haioun C, Chaumette MT (1995). Efficacy of single-agent chemotherapy in low-grade B-cell mucosa-associated lymphoid tissue lymphoma with prominent gastric expression. J Clin Oncol.

[23] Schechter NR, Portlock CS, Yahalom J (1998). Treatment of mucosa-associated lymphoid tissue lymphoma of the stomach with radiation alone. J Clin Oncol.

[24] Cogliatti SB, Schmid U, Schumacher U (1991). Primary B-cell gastric lymphoma: a clinicopathological study of 145 patients. Gastroenterology.

[25] Taal BG, Boot H, van Heerde P, de Jong D, Hart AA, Burgers JM (1996). Primary non-Hodgkin lymphoma of the stomach: endoscopic pattern and prognosis in low versus high grade malignancy in relation to the MALT concept. Gut.

[26] Pinotti G, Zucca E, Roggero E (1997). Clinical features, treatment and outcome in a series of 93 patients with low-grade gastric MALT lymphoma. Leuk Lymphoma.

[27] Montalban C, Castrillo JM, Abraira V (1995). Gastric B-cell mucosa-associated lymphoid tissue (MALT) lymphoma: clinicopathological study and evaluation of the prognostic factors in 143 patients. Ann Oncol.

[28] Ferrucci PF, Vanazzi A, Grana G (2007). High activity 90Y-ibritumomab tiuxetan (Zevalin) with peripheral blood progenitor cells support in patients with refractory/resistant B-cell non-Hodgkin lymphomas. Br J Haematol.

